# Near-shore Antarctic pH variability has implications for the design of ocean acidification experiments

**DOI:** 10.1038/srep09638

**Published:** 2015-04-09

**Authors:** Lydia Kapsenberg, Amanda L. Kelley, Emily C. Shaw, Todd R. Martz, Gretchen E. Hofmann

**Affiliations:** 1 Department of Ecology Evolution and Marine Biology, University of California Santa Barbara, Santa Barbara, California, 93106, United States of America; 2 Biophysical Remote Sensing Group, School of Geography, Planning and Environmental Management, The University of Queensland, Brisbane, Queensland, 4072, Australia; 3 Scripps Institution of Oceanography, University of California San Diego, La Jolla, California, 92093, United States of America

## Abstract

Understanding how declining seawater pH caused by anthropogenic carbon emissions, or ocean acidification, impacts Southern Ocean biota is limited by a paucity of pH time-series. Here, we present the first high-frequency in-situ pH time-series in near-shore Antarctica from spring to winter under annual sea ice. Observations from autonomous pH sensors revealed a seasonal increase of 0.3 pH units. The summer season was marked by an increase in temporal pH variability relative to spring and early winter, matching coastal pH variability observed at lower latitudes. Using our data, simulations of ocean acidification show a future period of deleterious wintertime pH levels potentially expanding to 7–11 months annually by 2100. Given the presence of (sub)seasonal pH variability, Antarctica marine species have an existing physiological tolerance of temporal pH change that may influence adaptation to future acidification. Yet, pH-induced ecosystem changes remain difficult to characterize in the absence of sufficient physiological data on present-day tolerances. It is therefore essential to incorporate natural and projected temporal pH variability in the design of experiments intended to study ocean acidification biology.

The extensive effects of ocean acidification, the systematic reduction of ocean pH due to the absorption of anthropogenic carbon dioxide (CO_2_) by surface oceans^1^, are predicted to be first observed in high-latitude seas^2^. Cold waters of the Southern Ocean are naturally rich with CO_2_, which results in low carbonate (aragonite and calcite) saturation states^2^. As ocean acidification progresses, pH and aragonite saturation state (Ω_arag_) will decrease and facilitate the dissolution of marine calcium carbonate. From a biological perspective, evolution in the absence of shell-crushing predators in the near-shore Antarctic has left many benthic biogenic calcifiers with relatively brittle shells^3^ that may be vulnerable to ocean acidification. Shell dissolution in live Southern Ocean pteropods, *Limacina helicina antarctica*, has already been observed in CO_2_-rich upwelled waters (Ω_arag_ ≈ 1)^4^. Antarctic marine biota is hypothesized to be highly sensitive to ocean acidification^5^, and predicting the impact of this anthropogenic process and the potential for future organismal adaptation is a research priority^6^.

To predict how future ocean acidification will affect any marine ecosystem, it is first necessary to understand present-day pH variability. In the Southern Ocean, there are strong seasonal cycles in carbonate chemistry^7^,^8^,^9^ due to the temporal partitioning of summertime primary production and wintertime heterotrophy^10^. Summertime phytoplankton blooms regularly drive the partial pressure of CO_2_ in seawater (pCO_2_) well below atmospheric equilibrium and are the primary source for pCO_2_ variability in the Southern Ocean^9^. This seasonal carbonate chemistry cycle corresponds to a summertime pH increase of 0.06 units on a regional scale in the Southern Ocean^11^ and as much as 0.6 units locally in Prydz Bay^7^ and the Ross Sea^12^. The summertime pH increase (e.g. 0.6) can thus exceed the 0.4 pH unit magnitude of ocean acidification predicted for 2100^13^.

Future ocean carbonate chemistry remains challenging to predict due to other environmental processes and biological feedbacks^14^. Southern Ocean aragonite undersaturation (approx. pH ≤ 7.9) is predicted to occur first during the winter season in the next 20 years^11^. However, seasonal ice cover may delay the onset of ocean acidification thresholds by a few decades due to reduced air-sea gas exchange^12^. Likewise, decreasing seasonal ice cover, due to changes in wind and air temperature, are estimated to yield at least a 14% increase in primary production in the Ross Sea by 2100^15^. This could potentially increase pH and Ω_arag_ in summer. Furthermore, increased stratification in the future may result in phytoplankton community shifts^15^. As an example, diatom communities dominate periods of highly stratified waters in the Ross Sea but drawdown less CO_2_ compared to the dominant bloom algae *Phaeocystis antarctica* that proliferate in deeply mixed waters^16^. Thus, seasonal changes in carbonate chemistry (for example, from primary production) may yield alternative scenarios for ocean acidification outcomes^17^. Currently, projections of ocean acidification for near-shore Antarctica are largely based on discrete sampling^11^, which may not have detected sub-seasonal (e.g. daily, weekly) pH variability that could be important for biological processes.

Although ocean acidification is generally predicted to be deleterious to marine life, not all taxa and species respond similarly to future conditions^18^. There is emerging evidence that an organism's pH-exposure history can influence its tolerance of ocean acidification. For example, Ref. 19 showed that an Arctic copepod species that experienced varied depth-dependent pH exposure was more tolerant of CO_2_-acidified seawater treatments compared to another Arctic copepod species that experiences a smaller range in pH. Comprehensive characterization of the ‘pH-seascape’ is thus necessary to link CO_2_-perturbation experiments with present-day and future organismal performance in the field. Such field time-series are sparse in near-shore Antarctica and are either extremely short^20^,^21^ or low in sampling frequency^7^,^8^,^12^.

In this study, our main goal was to describe pH variability experienced by organisms in near-shore Antarctica across seasonal transitions in an area with annual sea ice cover. In addition, we use the data to explore how pH variability and changes in seasonal CO_2_ drawdown (as a proxy for changes in primary production) may impact future trajectories of ocean acidification in our study region.

## Results

### pH data

**Figure 1:**
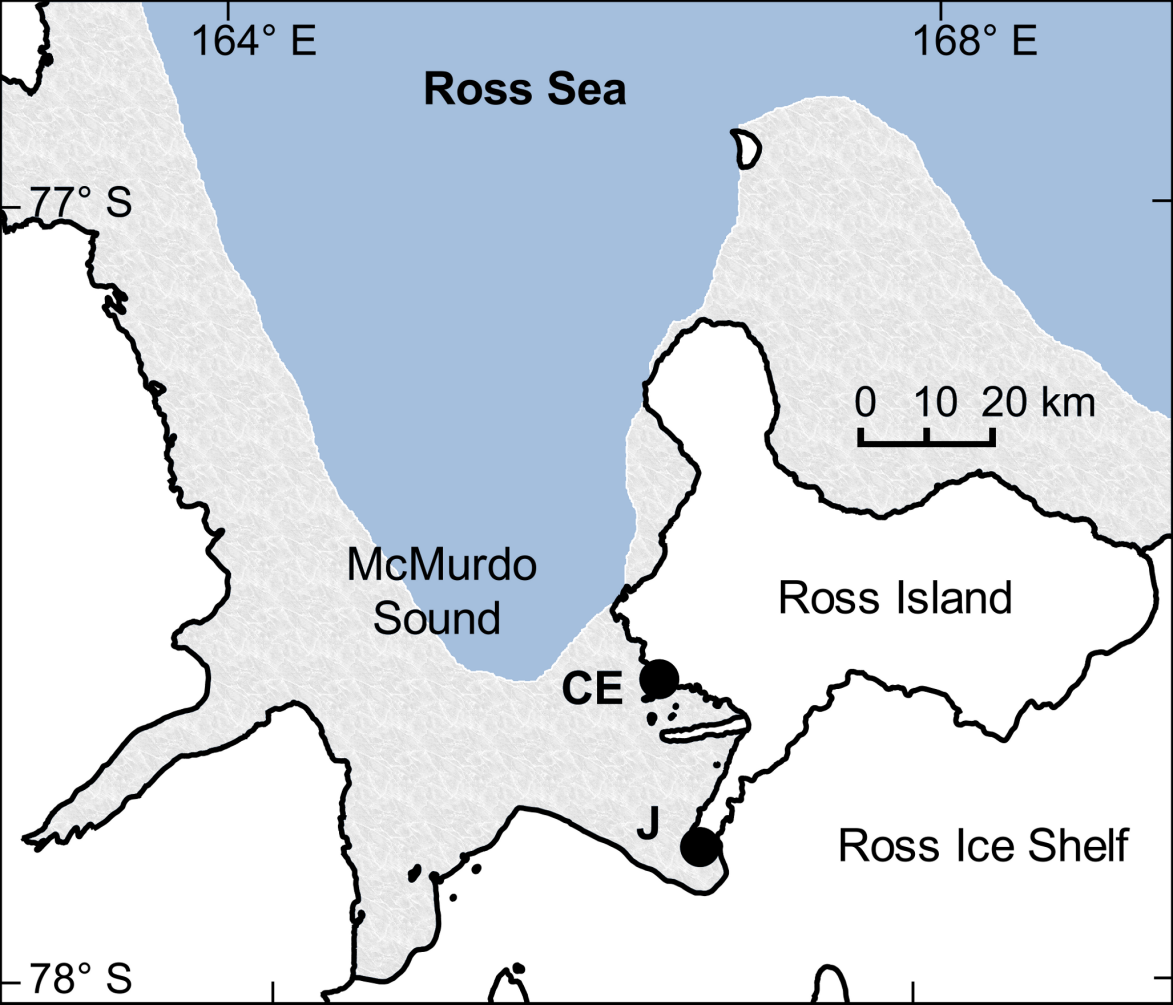
Map of pH sensor deployments in McMurdo Sound, Antarctica. Sensors were deployed at the Jetty (J) in 2011 and at Cape Evans (CE) in 2012. Annual sea ice contour (marble color) approximates November conditions for 2011 (RISCO Rapid*Ice* Viewer). Mapping data are courtesy of the Scientific Committee on Antarctic Research, Antarctic Digital Database. Map was constructed in QGIS (Version 2.0.1) and sea ice contour was added using GIMP (Version 2.6.11).

**Figure 2:**
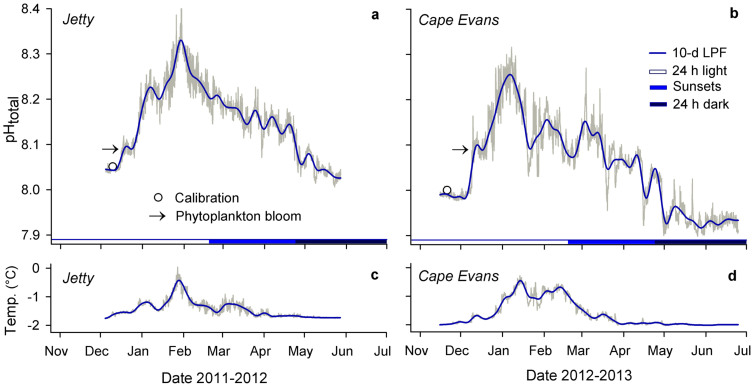
pH and temperature cycles in McMurdo Sound, Antarctica. Time-series pH (a, b) and temperature (c, d) at the Jetty and Cape Evans as recorded by SeaFET pH sensors (grey line). A 10-day low-pass filter (10-d LPF) was applied to the pH and temperature observations (blue line). Daylight is noted by colored x-axis bars where ‘sunsets’ indicates decreasing day length. Arrows indicate anecdotal events of phytoplankton blooms as observed by United States Antarctic Program SCUBA divers. Calibration samples are noted (circle). Ticks on x-axes denote the first day of the month.

**Figure 3:**
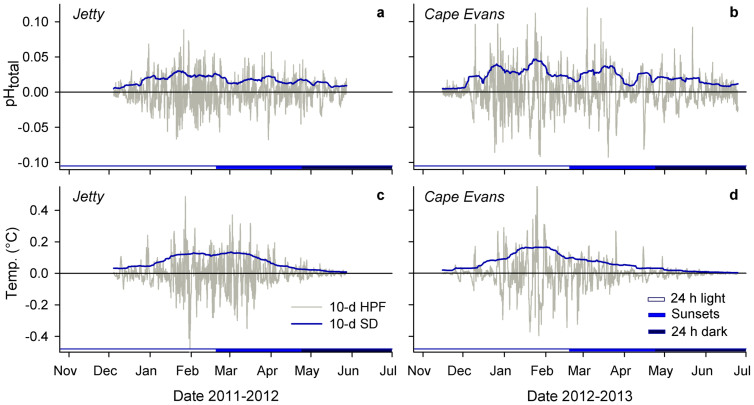
Seasonal increase in short-term pH and temperature variability. High-pass filtered pH (a, b) and temperature (c, d) at the Jetty and Cape Evans (10-day, 10-d HPF). Blue lines are the s.d. of a 10-day moving average on the high frequency data (grey line). Daylight is noted by colored x-axis bars.

To collect high-resolution pH data, we deployed autonomous SeaFET pH sensors^22^ in the austral spring at two sites in near-shore McMurdo Sound, Jetty and Cape Evans, on subtidal moorings in separate years (Fig. 1). Both the Jetty and Cape Evans showed four general sequences of pH variation during the observed period (Fig. 2a, 2b; Table 1). First, pH (reported on the total hydrogen ion scale for all measurements) rapidly increased from approximately 8.0 to 8.3 units in the early austral summer from December to January. Second, monthly pH variability from December through April (s.d. ± 0.03 to 0.08 units) was higher than that observed in November (s.d. ± 0.01 units). The increase in short-term pH variability remained after removing low-frequency pH variability that was inherently included in the monthly standard deviations listed in Table 1. Standard deviation of the 10-day moving average of high-pass filtered pH data was greater during the summer months relative to November, May, and June and peaked in January at both sites (<0.05 pH units, Fig. 3a, 3b). Third, following peak pH in January, pH and short-term pH variability generally declined to the end of April, but remained higher than November and early-December conditions. Fourth, around the onset of 24 h darkness at the end of April and during stabilized temperature pH declined and was followed by lower mean monthly pH and variability in May (s.d. ± 0.02 units) and in June (s.d. ± 0.01 units), relative to summer months. The initial pH increase from fall to peak January conditions corresponded to a decline in the calculated dissolved inorganic carbon (DIC) of 167 and 137 *µ*mol kgSW^−1^ at the Jetty (54 days) and Cape Evans (31 days), respectively.

The seasonal pH range was 0.30 and 0.33 pH units for the Jetty and Cape Evans, respectively (Fig. 1a, 1b), based on a 10-day low pass filter. Short-term pH variability contributed to a total range of observed pH from summer to winter conditions of 0.40 and 0.42 units, at the Jetty and Cape Evans, respectively. Maximum pH was observed in January and minimum pH was observed in May at both sites. Mean pH differed between the Jetty and Cape Evans when comparing pH observations of the same date range (8.15 ± 0.08 at the Jetty; 8.08 ± 0.09 at Cape Evans; Mann-Whitney Wilcoxon test, *p* < 0.001, *W* = 3481992, *n* = 2103). In general, summertime sub-seasonal (Fig. 2a, 2b) and short-term pH variability (Fig. 3a, 3b) was greater at Cape Evans in 2013 compared to the Jetty in 2012. Changes in pH of ± 0.13 units occurred various times over the course of hours to a day at Cape Evans. The largest pH change over a relatively short period was −0.27 units over 5.5 days in March at Cape Evans.

Within the same site, temperature data showed similar patterns in variability as pH: temperature increased from the start of the recording period, peaked in January, after which it declined and stabilized in early April to similar temperatures observed in November and early December (Fig. 2c, 2d). Low-pass filtered data show a seasonal warming of 1.33°C and 1.55°C at the Jetty and Cape Evans, respectively. Like pH, high-pass filtered temperature data showed a seasonal increase in short-term variability from January through April (Fig. 3d, 3d). Absolute seasonal temperature change was 1.8°C and 1.7°C at the Jetty and Cape Evans, respectively.

At both the Jetty and Cape Evans, temperature was significantly and positively correlated with pH over the deployment period (*p* < 0.001; Table 2), opposing the thermodynamic relationship. High-pass filtered temperature was significantly correlated with pH at both sites (Table 2), but the direction of this relationship was different at both sites and explains little of the overall pH variation (< 5%, Table 2).

When used for carbonate calculations (DIC, pCO_2_, Ω_arag_), pH data indicate that McMurdo Sound is currently supersaturated with respect to aragonite (Table 1). Monthly mean Ω_arag_ in late fall and early winter approached 1. Conditions may have actually reached undersaturation (Ω_arag_ <1) for brief periods at Cape Evans in May and June (minimum of Ω_arag_ 0.96), depending on the error in pH measurements (see Methods).

### Ocean acidification scenarios

**Figure 4:**
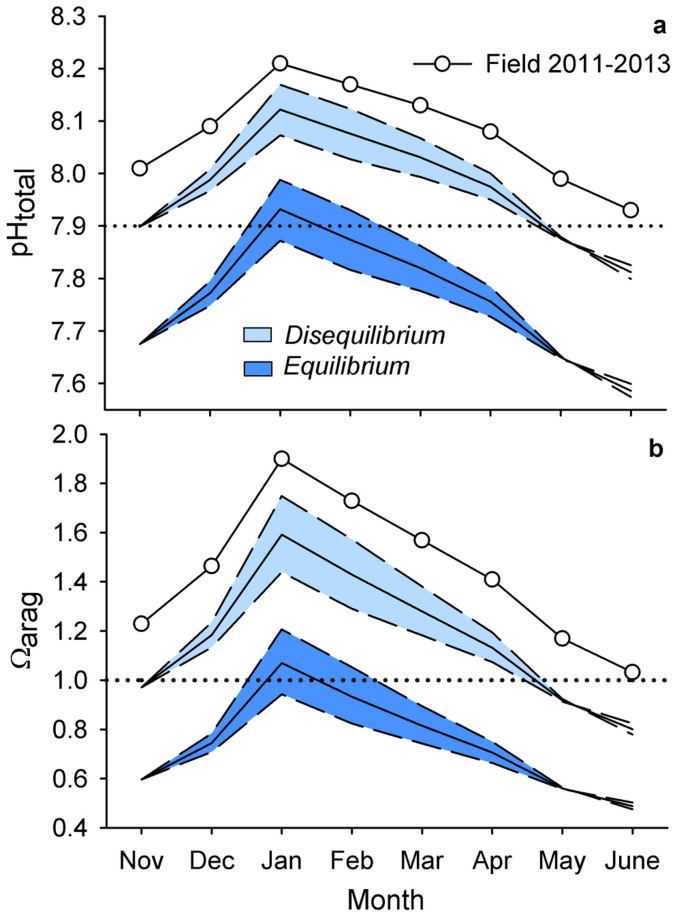
Present-day and end-century pH and aragonite saturation state. Present–day (circle) and end-century monthly mean pH (a) and aragonite saturation state, Ω_arag_ (b), in McMurdo Sound, Antarctica, using a disequilibrium and equilibrium scenario (solid line). Within each scenario, a simulated 20% increase (upper dashed lines) and decrease (lower dashed lines) in seasonal DIC amplitude is used to simulated changes in net community production. Dotted lines reference pH 7.9 and Ω_arag_ of 1.

**Figure 5:**
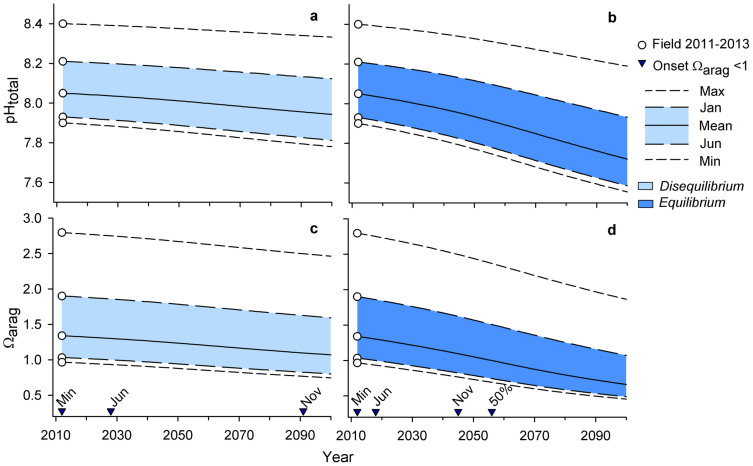
Annual changes in pH and aragonite saturation state ranges. Projections of yearly changes in pH and aragonite saturation state, Ω_arag_, in McMurdo Sound, Antarctica, using a disequilibrium (a, c) and equilibrium (b, d) scenario. Annual range in pH increases and Ω_arag_ decreases with future acidification. End-century maximum pH and Ω_arag_ remain above acidification thresholds of pH 7.9 and Ω_arag_ of 1. Projections are based on field data collected in 2011–2013 (circle). January and June monthly means represent mid-summer and winter conditions, respectively. The overall mean represent mean values from spring into winter conditions. Onset of aragonite undersaturation (triangles) is marked for each parameter and additionally for November monthly mean conditions.

McMurdo Sound regional ocean acidification trajectories were made using averaged pH observations from 2011–2013 and forced with the Representative Concentration Pathway 8.5 (RCP8.5) CO_2_ emission scenario^23^. Due to the potential offset in pH measurements associated with use of unpurified m-cresol dye (~0.03 pH units, see Methods), our results may slightly overestimate acidification trends. The equilibrium scenario^12^, which represents an increase in seawater pCO_2_ that tracks atmospheric levels, predicted more extreme acidification than the disequilibrium scenario^12^, which represents a 65% reduced CO_2_ uptake due to seasonal ice cover (Fig. 4, 5).

In both scenarios, CO_2_ forcing increased the seasonal pH amplitude and reflects the process of reduced ocean buffer capacity as CO_2_ is absorbed^24^. For example, present-day range of observed monthly mean pH from January to June was 0.28 units and increased to 0.31 and 0.35 units under the disequilibrium and equilibrium scenario, respectively. For all scenarios, wintertime pH of ~7.9 (approximate aragonite undersaturation) occurred by the end of the century (Fig. 4, 5). Assuming that pH < 7.9 persists for the period that we lack data for (July through October), the disequilibrium and equilibrium models suggest a 7- and 11-month annual duration of pH conditions < 7.9 units and undersaturation by 2100, respectively.

As a proxy for simulating changes in net community production, DIC amplitude was perturbed by ± 20% (Fig. 4). A 20% increase in seasonal DIC amplitude raised pH and Ω_arag_ during the summer and fall but failed to raise pH and Ω_arag_ to present-day levels. For example, under the equilibrium model, a 20% increase in seasonal DIC amplitude marginally extended end-century duration of summertime pH > 7.9 from January (pH 7.93, Ω_arag_ 1.07) to January (pH 7.99, Ω_arag_ 1.21) and February (pH 7.93, Ω_arag_ 1.05).

Any reduction in the amplitude of seasonal DIC will exacerbate the effects of ocean acidification. For example, during the month of peak pH, mean January pH remained above 7.9 units in all scenarios, except under the equilibrium scenario with a simulated 20% reduction in seasonal DIC amplitude (January pH 7.87, Fig. 4). This latter scenario was the only scenario that exhibited permanent aragonite undersaturation in McMurdo Sound by 2100.

Due to the increase in pH variability observed during summer months (Fig. 3), organisms at our study sites will likely still periodically experience pH > 7.9 and Ω_arag_ > 1 by 2100 (Fig. 5). For instance, under the equilibrium scenario, maximum pH was pH 8.19, and 0.47 units above mean January conditions (pH 7.72). Acidification thresholds (pH ~7.9 and Ω_arag_ < 1) were crossed earlier under the equilibrium model compared to the disequilibrium model (Fig. 5). Here, onset of June (i.e. winter) undersaturation was projected to occur by 2018, a decade earlier than under the disequilibrium scenario. November (i.e. spring) aragonite undersaturation was predicted to first occur by 2045 in the equilibrium model, 46 years earlier than predicted by the disequilibrium model. Timing of the threshold crossings may be delayed given the potential offset in Ω_arag_ associated with the pH measurement error.

## Discussion

The observed pH regime in McMurdo Sound can be grouped into two seasonal patterns: (1) stable pH with low variability during the winter and spring, and (2) elevated pH with high variability during the summer and fall. While our pH sensors did not record data from July through October, previous studies of pH (in October^21^) and temperature^25^ in this region support our hypothesis of low environmental variability during the winter. Note, observations from Prydz Bay^8^ (68°S) suggest that pH may decline slightly (<0.1 units) from June to September.

The amplitude of summertime pH elevation (0.3–0.4 units) observed in McMurdo Sound is among one of the greatest observed in the ocean and matches pH cycles at a northern coastal site in Prydz Bay, Antarctica^7^,^8^. In McMurdo Sound, the intense summertime DIC drawdown started in December and matched the timing of the annually recurring *Phaeocystis* sp. phytoplankton blooms, which are well-described and typically centered on 10 December (R. Robbins pers. comm.)^26^. The initial pH increase at Cape Evans (pH 8.01 to 8.12) occurred within 24 h on 9 December 2012 during which SCUBA divers noted sudden increase in phytoplankton presence in McMurdo Sound (R. Robbins pers. comm.).

Given that (1) the sudden increase in pH at our study sites followed a period of extremely stable pH conditions^20^,^21^, (2) maximum observed pH corresponded to pCO_2_ ~200 *μ*atm below atmospheric equilibrium, and (3) productive waters from the Ross Sea are advected south into east McMurdo Sound^10^, the initial rapid pH increase in December is likely the signature of phytoplankton blooms that originated in the Ross Sea and reached our coastal sites. Calculated DIC drawdown from fall to summer at the Jetty and Cape Evans (167 and 137 *µ*mol kgSW^−1^ DIC) matches the timing and magnitude of CO_2_ cycles observed at similar depths in the Ross Sea^27^ and Prydz Bay (~135–200 *μ*atm kgSW^−1^ DIC)^7^,^8^.

Following the peak pH in January, pH steadily declined to pre-summer conditions by the end of April. A recent study of autonomous pCO_2_ measurements on incoming seawater at Palmer Station from Arthur Harbor (64°S) observed a summertime increase in primary production, starting in November^28^. Here, a phytoplankton bloom was captured with peak production corresponding to an observation of 50 *μ*atm pCO_2_. Contrary to the slow return of carbonate chemistry to pre-summer conditions observed in McMurdo Sound over 4–5 months, pCO_2_ at Arthur Harbor rapidly returned to atmospheric equilibrium in December and persisted to the end of the study in March. The authors attributed the crash of the bloom to physical mixing and zooplankton grazing, which would control phytoplankton density and contribute respiratory CO_2_. Depending on the year-to-year pH variability on the Antarctic Peninsula, the season of high pH in Arthur Harbor may potentially be much shorter compared to that in McMurdo Sound. For example, interannual carbonate chemistry variability in the Weddell Sea is linked to the timing of sea-ice melt and phytoplankton productivity in the mixed layer^29^. The decline in pH observed at McMurdo is likely a combination of reduced primary production, increased heterotrophy and deepening of the mixed layer, as has been suggested to occur in Prydz Bay^7^ and observed in other notable bloom regions such as the North Atlantic^30^.

Calculated pCO_2_ at Cape Evans in April (403 ± 44 *μ*atm) nears observations from the Ross Sea made in April 1997 (320–400 *μ*atm)^9^. A stabilization of pH in May and June at Cape Evans corresponded to ~500 *μ*atm pCO_2_. We were unable to collect validation samples during this period, however, biofouling was not an issue at our sites and SeaFET pH sensors have been shown to maintain stability over >9 months^31^. Similar observations have been made elsewhere in near-shore Antarctica. For example, high pCO_2_ (~490 *μ*atm) was observed near the Dotson Ice Shelf in the Amundsen Sea Polynya in summer and was correlated with the deepening of the mixed layer relative to the surrounding area^32^. In addition, the range of Ω_arag_ from mean summer (January) to winter (May) conditions was 0.70 and 0.75 at the Jetty and Cape Evans, respectively, matching the latest observations from Prydz Bay (0.73^8^) and the Weddell Sea (0.77^29^). The low pCO_2_ recorded in May and June at Cape Evans may thus be a combination of water column mixing and heterotrophy, as well as a potential 37 *μ*atm pCO_2_ overestimation associated with the offset of our pH measurement.

The observed ~0.3 unit summertime increase in pH in McMurdo Sound is much larger than that of northern high-latitudes^33^. While primary productivity in Antarctic waters is comparable to that of the high-latitude North Atlantic and Pacific^9^, the observed < 2°C annual temperature variation is typical of McMurdo Sound^25^ and plays almost no role in the seasonal amplitude of pH (1.8°C warming corresponds to a pH decrease of 0.03 units). In contrast, at locations such as the North Pacific the temperature cycle can be ~5 times greater than the observed range of temperatures in this study^9^. At our sites, the seasonal temperature forcing on pH counteracts seasonal forcing by primary production. As a result, the absence of a significant temperature forcing in near-shore Antarctica leads to a more pronounced seasonal pH cycle with greater amplitude compared to other bloom regions in the world^33^.

As captured in our dataset, the summer season in McMurdo Sound is marked by an increase in sub-seasonal and short-term pH variability from December through April. In terms of s.d. of unfiltered (monthly s.d.) and high-pass filtered (10-day s.d.) pH, pH variability in McMurdo Sound is of similar magnitude to that observed in temperate kelp forests (e.g. ± 0.043 − 0.111) and tropical coral reefs (e.g. ± 0.022) over 30 days^34^. This is surprising due to absence of large temperature forcing and structural macrophytes and holobionts, which induce diurnal pH cycles at lower latitudes. On a Hawaiian reef, variability in pH was correlated with environmental parameters such as wave and height, wind speed, and solar radiation^35^, suggesting a combination of influential abiotic and biotic drivers on coastal seawater pH variability.

We did not directly measure abiotic and biotic factors that influence carbonate chemistry in our study region and more measurements would be needed to quantify the sources of variability over different frequencies. For instance, air-sea gas exchange contributes to pH on a seasonal timeframe, where summertime CO_2_ uptake by the ocean during ice-free periods masks the total contribution of net community production to DIC drawdown^7^. Likewise, summer meltwater dilutes DIC and A_T_^7^,^8^ and may contribute to short-term pH variability in summer. The timing of sea ice melt onset may impact the duration and magnitude of carbonate chemistry seasonality where early melting enhances phytoplankton production under optimal mixed layer depths, as has been observed in the Weddell Sea^29^. Small pH variability (8.009 ± 0.015) observed from late October through November in McMurdo Sound may be explained by algal photosynthesis, although tides may play a small role as well^36^. Tidal exchanges of shallow and deeper water masses could play a larger role in summer pH variability, compared to spring^36^, when the water column is highly stratified^37^. Low pH variability observed in winter and spring could also stem from a decrease in respiratory CO_2_ contributions to DIC due to metabolic depression during periods of low food availablity, as has been observed to occur in pteropods^38^. In contrast, increased pH variability during the summer and fall is potentially influenced by the dominant biological forcing on the carbonate system in the Ross Sea at that time^9^. Such phytoplankton blooms create large spatial differences in pCO_2_^32^ that could lead to sub-seasonal and short-term pH variability through bloom patchiness across water mass movement. Quantification of abiotic and biotic parameters described above would improve estimations for future ocean acidification when incorporated into sensitivity models^17^.

We explored how seasonal pH variability may influence future ocean acidification in our study region in order to provide guidelines for biological experiments assessing future species' and ecosystem responses. The equilibrium and disequilibrium models provide boundaries for potential worst- and best-case acidification under a CO_2_ emission scenario that does not account for climate mitigation efforts^23^. Within all model parameters we employed, marine biota at our study sites are anticipated to experience changes beyond the envelope of current conditions, as has been predicted for lower latitude marine ecosystems as well^17^. As atmospheric CO_2_ continues to increase, (1) pH and duration of summertime high pH (> 7.9) will decrease and (2) the magnitude of seasonal and short-term pH variability may increase.

Previous studies of ocean acidification in the Southern Ocean and the Ross Sea identify the importance of seasonality and predict onset of wintertime aragonite undersaturation (Ω_arag_ < 1) between 2030 and 2050 under Intergovernmental Panel on Climate Change emissions scenario IS92a^2^,^11^,^12^. Our calculations of Ω_arag_ show wintertime undersaturation in McMurdo Sound occurring within this same timeframe, despite the higher CO_2_ emission scenario and high-resolution data used in our study, and potential over estimation of acidification trends associated with the offset in pH measurements. Given that pH and Ω_arag_ may decrease slightly from June through September^8^ and the lack of pH observations during these months, it is possible that periodic aragonite undersaturation may occur sooner than our predictions based on June observations. For context, the consequences of such periodic undersaturation could lead to calcium carbonate dissolution of live animals, as was observed for *L. helicina antarctica* at Ω_arag_ ≈ 1^4^. Likewise, studies on Antarctic sea urchin, *Sterechinus neumayeri*, early development conducted during the period of stable spring pH and urchin spawning in McMurdo Sound, suggest that persisting conditions of pH < 7.9 (approximate aragonite undersaturation) may to impair larval growth^39^ and calcification (G. E. Hofmann and P. C. Yu, unpubl.). Such conditions could occur in the latter half of this century during the sea urchin spawning season. Future carbonate chemistry conditions will ultimately depend on the rate at which anthropogenic CO_2_ is released to the atmosphere and any future changes in local physical and biological processes that our model does not account for (e.g. changes in temperature, meltwater, wind, mixing and stratification, upwelling, gas-exchange, and phytoplankton blooms).

Despite the dominant biological footprint in pH seasonality in the Southern Ocean, a 20% increase in seasonal DIC amplitude (simulating an increase in net community production) failed to raise pH to present-day levels at our study site. This suggests that relatively large changes in seasonal primary productivity may have a small effect on the pH exposure of coastal organisms relative to the changes induced by ocean acidification. Phytoplankton blooms, as a food source however, may impact species responses to ocean acidification. For example, a study of *L. helicina antarctica* collected in McMurdo Sound found that (1) feeding history (e.g. weeks, months, seasons) impacted oxygen consumption rates and (2) metabolic suppression due to low pH exposure was a masked during periods of food limitation^38^. This study highlights the importance of incorporating environmental history when interpreting experimental results. As the feeding history is likely correlated with pH exposure in the bloom, parsing out the effects of pH history and food availability will present a challenge for Antarctic physiology.

In Antarctic ocean acidification biology, ‘control’ conditions used in experiments are often ∼pH 8.0 (e.g. Ref. 39, 40) and represent current spring conditions in McMurdo Sound. Based on our future projections, this ‘control’ treatment will only occur during summer months if at all. Regardless of the exact rate of ocean acidification, the seasonal window of pH > 7.9 and Ω_arag_ > 1 will likely shorten in the future. This shrinking and seasonally shifting window of high pH may lead to unpredictable ecological consequences through changes in physiological and seasonally dependent biological processes (e.g. sea urchin larval development). It remains largely unknown how summertime pH levels currently contribute to animal physiology and whether or not a reduction in future peak pH and duration of high pH exposure influences physiological recovery following 7–11 months unprecedented low pH conditions. As an example, oxygen consumption and gene expression of heat shock protein 70 in the Antarctic bivalve *Laternula elliptica* increased when adults were exposed to experimental conditions near the habitat maxima (pH 8.32, categorized as ‘glacial levels’ by the authors) and below their current pH exposure (pH 7.77), relative to performance at ∼pH 8.0^40^. These results suggest that summer exposures may induce stress similar to conditions predicted with ocean acidification. Understanding how organisms are adapted to their present-day exposures will help elucidate how they will respond to future conditions.

As the exposure period of pH > 7.9 shrinks under simulated ocean acidification, the magnitude of annual pH variability increases. These changes suggest that calcifying marine biota of Antarctic coastal regions will experience larger seasonal pH cycles in addition to exposure to lower environmental pH. Due to the reduced buffer capacity of the ocean under high CO_2_, it is likely that the short-term pH variability in McMurdo Sound will be amplified in the future as well^24^. This has been predicted for coral reefs under ocean acidification scenarios^17^ and shown experimentally in pelagic field mesocosms^41^ where primary production drives diurnal pH cycles.

Our results provide guidance for the design of biological experiments aimed to address the potential for Antarctic species to adapt to a seasonally shrinking window of future high pH conditions. Although ocean acidification is likely to create an unprecedented marine environment, the existing presence of high pH variability in near-shore Antarctica may have beneficial implications for biological tolerance of ocean acidification. The distinct summertime increase in pH and pH variability in near-shore McMurdo Sound suggests that marine biota here have some capacity to deal with large fluctuations in the carbonate system, as has also been suggested by Ref. 42 in relation to the seasonal pH cycle. Unlike temperate upwelling regions where pH variability frequently drops below pH 8.0^43^, elevation of summer pH in McMurdo Sound opposes the direction of future ocean acidification. Future studies are necessary to describe how this pH-seascape may select for physiological tolerances of ocean acidification. For example, are natural positive (e.g. near-shore Antarctica) or negative deviations (e.g. temperate upwelling systems^43^) from pH 8.0 important for tolerance of future acidification? Will high summertime pH prepare organisms for low pH conditions in the winter? What frequency of pH variability promotes acidification tolerance?

A few recent studies have tackled such questions in temperate regions with mixed results. For example, Ref. 44 found that larval growth of mussel *Mytilus galloprovincialis* veligers was reduced under low static pH but recovered under similar conditions of low mean pH when semi-diurnal pH variability was introduced. However, congener *M. californianus* did not exhibit this ‘rescued’ response with diurnal cycles^44^. Although the Southern Ocean does not experience year-round diurnal photoperiods, a similar experimental approach can be used to guide studies on the impact of pH seasonality on ocean acidification tolerance^45^, and ultimately, adaptation.

We highlight a coupled oceanography and biology research strategy for studying ocean acidification biology in the Southern Ocean. Studying physiological tolerance and local adaptation to variable seawater chemistry ideally requires large differences in spatial and temporal pH variability^34^,^43^. If patterns of pH variability differ spatially around the Antarctic continent (e.g. McMurdo Sound vs. Arthur Harbor^28^), we can begin to investigate possible levels of adaptation to local pH regimes as a proxy for evolutionary adaptions to future conditions^46^. In other words, evidence of adaptation in space suggests that animals may be able to adapt in time, as the capacity to do so is linked directly to standing genetic diversity in populations^47^. As illustrated in the Southern Ocean, population level differences (e.g. Ross Sea vs. Western Antarctic Peninsula biota) and local adaptation in tolerance of future anthropogenic stressors may be possible due to different rates in regional warming^48^. Some studies have shown genetic structure across the biogeographic boundary of the Drake Passage (reviewed by Ref. 49). Studies regarding population differences in pH tolerances and exposures in circum-Antarctic species can be accomplished with strategic placement of oceanographic sensors and design of biological experiments with environmentally relevant pH treatments^43^,^50^. In addition, use of autonomous pH sensors would address the need for pH observations at high-latitudes^5^,^38^.

## Methods

### Study sites and deployment

Autonomous SeaFET pH sensors containing Honeywell DuraFET® electrodes^22^ were deployed in the austral spring at two sites in separate years on subtidal moorings in near-shore east McMurdo Sound (Fig. 1). Two SeaFETs were deployed side-by-side in December 2011 at a site near McMurdo Station (the Jetty, -77.85115, 166.66425), and one SeaFET was deployed during November 2012 at Cape Evans (-77.634617, 166.4159). Cape Evans is located 25 km north of the Jetty and is a highly productive site with an abundance of fish, macrophytes and marine invertebrates, including the sea urchin *S. neumayeri.* This site has previously been important for ocean acidification biology^20^,^39^. Subtidal moorings were anchored at approximately 27 m with sensor depth of 18 m. SeaFETs sampled on a two-hour frequency.

### Calibration

All reported pH is on a total hydrogen ion scale and listed as ‘pH’. Raw voltage recorded by the SeaFETs was converted to pH using one discrete seawater sample per sensor deployment following methods from Ref. 31. Calibration samples were collected via SCUBA following sensor conditioning to seawater within the first two weeks of each deployment, using a 5 L GO-FLO sampling bottle. Ideally, additional validation samples are collected throughout a sensor deployment. However, the remoteness of our sites restricted this work to one discrete sample per sensor deployment.

Calibration samples were preserved with saturated mercuric chloride according to Standard Operating Procedure (SOP) 1^51^. Spectrophotometric pH was determined at 25°C following SOP 6b^51^ using m-cresol purple from Sigma-Aldrich®. Total alkalinity (A_T_) was measured via open-cell titration with a Mettler-Toledo T50 (SOP 3b^51^). Salinity was measured using a calibrated YSI 3100 Conductivity Instrument. Certified Reference Materials of seawater (CRMs) and acid titrant were supplied by Dr. Andrew G. Dickson (University of California San Diego, Scripps Institution of Oceanography). pH at in situ temperature, as recorded by SeaFETs, was calculated from spectrophotometric measurements of pH_25°C_ and A_T_ and salinity on the bottle sample using the program CO2Calc [Version 1.0.1, 2010, U.S. Geological Survey] with CO_2_ constants from Ref. 52 refit by Ref. 53. All reported carbonate system calculation were conducted according to these constants.

### Data processing and analysis

Raw data from the SeaFETs were cropped based on battery exhaustion, which occurred before sensor recovery. One of the two sensors deployed at the Jetty failed quality control analyses, and data from this instrument are not reported. Inspections of raw voltages recorded by the functional SeaFETs confirmed that the calibration samples were collected after the period of sensor conditioning to seawater. In the absence of biofouling (as was the case for our sensors), sensor stability has been demonstrated over similar deployment times^31^ thereby generating high-quality pH datasets. A comparison of pH from each site was conducted using a Mann-Whitney Wilcoxon test as pH values were not normally distributed (Minitab® 16, Kolmogorov-Smirnov test, *p* < 0.10, for each site). All time is reported as UTC.

Time-series carbonate parameters were calculated from pH measurements using CO2calc for a depth of 18 m. Monthly mean salinity data was used from prior measurements in McMurdo Sound^54^ (Table 3). A_T_ was calculated from the empirical relationship between sea surface salinity (SSS) and sea surface temperature (SST, as measured by SeaFETs) for the Southern Ocean as reported by Ref. 55: 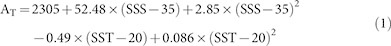


A_T_ measurements on SeaFET calibration samples matched the calculated A_T_ within the accuracy of titrator (A_T_ and salinity were 2342 *µ*mol kgSW^−1^ and 34.3 for the Jetty; 2351 *µ*mol kgSW^−1^ and 34.6 for Cape Evans, respectively). Monthly mean nutrient concentrations were estimated from the literature for McMurdo Sound and various Ross Sea stations in close proximity following the directions of ocean currents (max measurements month^−1^ = 4). Due to the lack of published phosphate measurements for this region, the Redfield ratio was applied to estimate phosphate from nitrate and silicic acid concentrations, in some cases (W. O. Smith, Jr. pers. comm.).

Summertime decrease in DIC was calculated for both sites from stable fall mean DIC conditions to minimum DIC observed in summer. Temperature and pH data were analyzed for event-scale to seasonal (10-day low-pass filter) and short-term (10-day high-pass filter) trends. Standard deviation of a 10-day moving average window on high-pass filtered data was calculated to describe seasonal changes in short-term pH and temperature variability. Unfiltered and 10-day high-pass filtered pH and temperature data from the duration of the entire deployment was investigated for each site using a linear correlation analysis (Matlab R2012b, Minitab® 16).

### Error estimates

SeaFET thermistors were not individually calibrated resulting in a maximum estimated temperature error of ~0.3°C. The estimate of the combined standard uncertainty associated with the pH measurement of the calibration samples is ± 0.026 pH units (quadratic sum of partial uncertainties). The quantified sources in pH error are: use of unpurified m-cresol dye (0.02^56^), spatio-temporal mismatch of the calibration sample (± 0.015^31^), user differences (± 0.006), and calibration of the SeaFET thermistor (± 0.005). Measurements of spectrophotometric pH on CRMs, although not specified by the SOP, suggest that our benchtop methods may underestimate pH_25°C_ by 0.032 (± 0.006, *n* = 18, across different users and days) relative to theoretical CRM pH calculated from DIC, A_T_, and salinity. It is hoped that, in the future, purified indicator dye will become widely available to the oceanography community in order to improve accuracy of pH measurements. The estimated uncertainty for the pH of calibration samples does not impact the relative changes in pH recorded by the SeaFET on hourly to monthly time scales, which in the absence of biofouling can be resolved to better than 0.001. Thermistors provide a stable temperature reading with resolution of better than 0.01°C. Based on replicate analyses of CRMs, the precision of the titration system used for calibration samples is ± ≤10 *µ*mol kgSW^−1^ and did not impact the pH calculation of our calibration samples at in situ temperatures. Errors in salinity were not quantified. Instead, calculations of DIC, pCO_2_, and Ω_arag_ from the pH time-series were conducted using monthly estimates of A_T_ and salinity (Table 3). For reference, a +0.026 pH error corresponds to errors under November (January) conditions of -9 (-11) *µ*mol kgSW^−1^ DIC, -27 (-17) *µ*atm pCO_2_, and +0.07 (+0.10) Ω_arag_.

### Ocean acidification scenarios

RCP8.5, which predicts atmospheric CO_2_ to reach 935.87 ppm by 2100^23^, was used to generate four ocean acidification scenarios. The equilibrium scenario assumes an increase in DIC at the same rate as would be expected if seawater pCO_2_ tracks the atmospheric value (~100 *µ*mol kgSW^−1^ increase in DIC by 2100) and (2) the disequilibrium scenario assumes a DIC increase at a 65% slower rate due to seasonal ice cover^12^. Secondary simulations of a ± 20% change in the observed seasonal amplitude of DIC are included along with the CO_2_ forcing scenarios. The disequilibrium model likely overestimates pH and Ω_arag_ as horizontal advection of northern ice-free water masses with longer surface residence times was not accounted for Ref. 12.

First, November was used as a baseline for CO_2_ forcing scenarios because it is a period of stable pH and has been measured for three consecutive years at Cape Evans^20^,^34^,^36^. Based on these prior studies and data collected in November 2012 during this study, mean November pH from 2010–2012 was pH 8.01. Calculated mean November seawater pCO_2_ was then forced with pCO_2_ from the RCP8.5 emission scenario assuming air-sea equilibrium, and annual changes in pCO_2_ were used to calculate annual changes in November DIC up to 2100.

Second, monthly mean pH and temperature observations from the Jetty and Cape Evans from 2011–2013 were averaged to calculate a partial (8-month), present-day, regional DIC climatology. Calculations were performed in CO2calc following methods listed above, with the exception that monthly mean temperature in June at Cape Evans was corrected from −2.0°C up to −1.9°C to match previous long-term observations^25^. Input variables are listed in Table 3. Starting from the November baseline, present-day changes in DIC where calculated by month (December – June) and for the maximum and minimum observed DIC and overall mean. Monthly changes in DIC were assumed constant for future projections and were applied to end-century November DIC to generate a DIC climatology for 2100. Annual DIC trajectories were modeled for observed minimum and maximum DIC, November, January, June, and overall mean. For simulations of ± 20% change in seasonal DIC amplitude, monthly changes in DIC were increased or decreased by 20%. Owing to the lack of projections of future warming for coastal Antarctica, the effects of future temperature change were not included in our simulation.

## Additional information

**Accession codes:** Temperature and pH data are deposited on Biological and Chemical Oceanography Data System. Hofmann, GE. ‘‘pH temp sal.’’ BCO DMO, WHOI. iPub: 13 March 2015.

## Figures and Tables

**Table 1:** Carbonate parameters at two sites in McMurdo Sound, Antarctica

Site, year		pH	T (°C)	DIC (*µ*mol kgSW^−1^)*	pCO_2_ (*µ*atm)*	Ω_arag_*
**Jetty**	*mean*	8.15 ± 0.08	−1.45 ± 0.31	2179 ± 37	302 ± 62	1.68 ± 0.29
2011–2012	*median*	8.16	−1.50	2177	292	1.66
	*min*	8.01	−1.80	2058	152	1.22
	*max*	8.40	0.00	2238	428	2.81
	*range*	0.40	1.80	181	276	1.60
	*Dec*	8.08 ± 0.04	−1.54 ± 0.14	2216 ± 17	355 ± 36	1.45 ± 0.14
	*Jan*	8.24 ± 0.05	−1.08 ± 0.36	2142 ± 26	236 ± 31	2.03 ± 0.23
	*Feb*	8.23 ± 0.04	−1.28 ± 0.25	2138 ± 17	241 ± 23	1.95 ± 0.16
	*Mar*	8.17 ± 0.03	−1.42 ± 0.19	2170 ± 10	286 ± 19	1.70 ± 0.09
	*Apr*	8.12 ± 0.04	−1.67 ± 0.05	2191 ± 14	319 ± 31	1.55 ± 0.11
	*May*	8.05 ± 0.02	−1.73 ± 0.01	2224 ± 7	387 ± 20	1.33 ± 0.06
**Cape Evans**	*mean*	8.05 ± 0.10	−1.63 ± 0.47	2218 ± 39	391 ± 92	1.37 ± 0.30
2012–2013	*median*	8.06	−1.90	2216	372	1.36
	*min*	7.90	−2.04	2107	192	0.96
	*max*	8.32	−0.27	2276	559	2.34
	*range*	0.42	1.77	168	367	1.39
	*Nov*	7.99 ± 0.01	−1.96 ± 0.0	2256 ± 2	450 ± 6	1.17 ± 0.01
	*Dec*	8.09 ± 0.08	−1.73 ± 0.2	2212 ± 30	349 ± 67	1.49 ± 0.25
	*Jan*	8.17 ± 0.07	−0.90 ± 0.3	2170 ± 29	285 ± 53	1.79 ± 0.25
	*Feb*	8.11 ± 0.04	−0.96 ± 0.3	2183 ± 15	327 ± 31	1.56 ± 0.12
	*Mar*	8.10 ± 0.05	−1.75 ± 0.1	2198 ± 18	342 ± 42	1.46 ± 0.15
	*Apr*	8.03 ± 0.04	−1.95 ± 0.0	2225 ± 14	403 ± 44	1.27 ± 0.10
	*May*	7.94 ± 0.02	−1.99 ± 0.0	2262 ± 7	508 ± 29	1.04 ± 0.05
	*Jun*	7.93 ± 0.01	−2.00 ± 0.0	2268 ± 3	518 ± 18	1.03 ± 0.02

Error is ± s.d.

*Calculated parameter.

**Table 2:** Linear regression analysis of pH and temperature

Site	Predictor	Coef	SE Coef	*T*	*p*	*R^2^*
**Jetty**	*Temperature*	0.20526	0.00358	57.27	<0.001*	0.61
	*Temperature 10-d HPF*	−0.04749	0.00453	−10.49	<0.001*	0.05
**Cape Evans**	*Temperature*	0.14985	0.00275	54.51	<0.001*	0.53
	*Temperature 10-d HPF*	0.02912	0.0058	5.02	<0.001*	0.01

*Statistically significant.

10-d HPF, 10-day high-pass filtered data.

**Table 3:** Model inputs for seasonally variable parameters. See Methods for details

Month	pH	Temp. (°C)	Salinity^54^	Total Alkalinity (*µ*mol kgSW^−1^)∞	Total PO_4_ (*µ*mol kgSW^−1^)	Total Si (*µ*mol kgSW^−1^)
Nov*	8.01	−1.9	34.82	2348	1.9^10^,^37^,^57^,^58^	65.1^10^,^57^
*Dec*	8.09	−1.6	34.76	2343	1.3^10^,^57^,^59^	62.1^10^,^57^,^59^
*Jan*	8.21	−1.0	34.65	2335	0.9^10^,^57^,^59^	50.7^10^,^57^,^59^
*Feb*	8.17	−1.1	34.37	2322	1.3^59^	54.5^59^
*Mar*	8.13	−1.6	34.44	2327	1.5^57^	70.5^57^
*Apr*	8.08	−1.8	34.50	2331	2.1^12^	78.9^12^
*May*	7.99	−1.9	34.62	2337	2.1^12^	78.9^12^
Jun*	7.93	−1.9	34.69	2341	2.1^12^	78.9^12^
mean*	8.05	−1.6	34.65	2335	1.6	67.5
min*	7.90	−1.9	34.62	2337	2.1	78.9
*max*§	8.40	−0.4	34.61	2336	0.9	50.7

*pH and temperature data from Cape Evans only, based on data collected in this study and in Refs. 20,34,36.

§pH and temperature data from the Jetty only.

∞Calculated from salinity and temperature^55^.
